# Natural Neurobiological Active Compounds in Parkinson’s Disease: Molecular Targets, Signaling Pathways, and Therapeutic Prospects

**DOI:** 10.3390/ijms27031301

**Published:** 2026-01-28

**Authors:** Xue Wu, Linao Zhang, Shifang Luo, Qing Li, Jiying Wang, Wentao Chen, Na Zhou, Lingli Zhou, Rongyu Li, Yuhuan Xie, Qinghua Chen, Peixin Guo

**Affiliations:** 1 Yunnan Key Laboratory of Dai and Yi Medicine, Yunnan University of Chinese Medicine, Kunming 650500, China; 15752427670@163.com (X.W.); zla13975@163.com (L.Z.); 18608342484@163.com (S.L.); 15758174180@163.com (Q.L.); 15708676760@163.com (J.W.); a15859361020@163.com (W.C.); 18487143535@163.com (N.Z.); zhoulingli0220@163.com (L.Z.); 18053822920@163.com (R.L.); 13698798029@163.com (Q.C.); 2 College of Chinese Medicine, Yunnan University of Chinese Medicine, Kunming 650500, China; 3 College of Ethnic Medicine, Yunnan University of Chinese Medicine, Kunming 650500, China; 4 College of Basic Medical Sciences, Yunnan University of Chinese Medicine, Kunming 650500, China

**Keywords:** natural compounds, Parkinson’s disease, mechanism, traditional Chinese medicine

## Abstract

Parkinson’s disease (PD) is a progressive neurodegenerative condition with a multifactorial etiology, characterized by dopaminergic neurons being selectively absent in the midbrain. Clinically, PD manifests primarily with core motor symptoms of resting tremor, bradykinesia, and muscle rigidity, and is often accompanied by non-motor symptoms including depression, cognitive impairment, and gastrointestinal dysfunction. Among the extensive relevant research, few have explored the precise pathogenic mechanisms underlying PD, and no curative treatment is available. Current pharmacological therapies mainly provide symptomatic relief by enhancing central dopaminergic function or modulating cholinergic activity; however, their long-term efficacy is frequently constrained by waning therapeutic response, drug tolerance, and adverse reactions. Accumulating evidence suggests that several naturally derived neuroactive compounds—such as gastrodin, uncarin, and paeoniflorin—demonstrate significant potential in combating PD. In this systematic review, we examined original research articles published from 2010 to 2025, retrieved from PubMed, Web of Science, and CNKI databases, using predefined keywords of Parkinson’s disease, neuroprotective herbal compounds, traditional medicine, multi-target mechanisms, natural product, autophagy, neuroinflammation, and oxidative stress. Studies were included if they specifically investigated the mechanistic actions of natural compounds in PD models. Conference abstracts, review articles, publications not in English or Chinese, and studies lacking clearly defined mechanisms were excluded. Analysis of the available literature reveals that natural neuroactive compounds may exert anti-PD effects through multiple mechanisms, e.g., inhibiting pathological α-synuclein aggregation, attenuating neuronal apoptosis, suppressing neuroinflammation, mitigating oxidative stress, and restoring mitochondrial dysfunction. This review provides insights that may inform the clinical application of natural bioactive compounds and guide their further development as potential therapeutic candidates against PD.

## 1. Introduction

Parkinson’s disease (PD) represents a progressive and multifactorial neurological disorder featuring the selective and progressive loss of dopaminergic neurons [1]. One of its central pathological hallmarks is dopaminergic neuron degeneration within the substantia nigra (SN) [2], particularly within the ventrolateral layer, which contains neurons projecting to the dorsal shell of the striatum. Clinical-pathological correlation studies indicate that moderate to severe loss of dopaminergic neurons in this area may underpin the bradykinesia and rigidity characteristic of advanced PD [3,4]. Another defining feature of PD is the Lewy body pathology, which involves the accumulation of abnormally folded protein aggregates—a phenomenon observed across multiple neurodegenerative diseases, including PD [5,6]. In PD, this protein has been identified as α-synuclein (α-syn). When misfolded, α-syn loses solubility and aggregates within neuronal cell bodies, where it forms Lewy bodies, and in neuronal processes, where it gives rise to Lewy neurites. Clinically, PD manifests predominantly with motor symptoms of resting tremor, muscle rigidity, and bradykinesia, frequently accompanied by non-motor symptoms of depressed mood, cognitive impairment, and gastrointestinal dysfunction [7]. Epidemiological studies indicate that PD ranks as the second-most prevalent neurodegenerative disorder worldwide after Alzheimer’s disease (AD), which affects nearly 10 million individuals, and its incidence exhibits an uptrend annually. Owing to the lack of curative therapies and the complexity of symptom management, PD not only imposes a significant economic burden on healthcare systems, but also severely affects patients’ quality of life [8].

**Figure 1:**
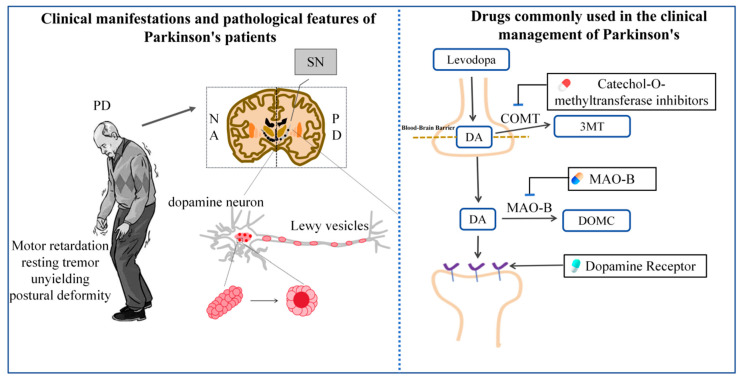
Pathological features of PD and commonly used clinical medications.

Modern pharmacological treatment for PD remains largely symptomatic, primarily achieved by enhancing central dopamine neurotransmission or reducing cholinergic neurotransmission [9]. First-line therapeutic agents include levodopa, selegiline, amantadine, and anticholinergic drugs such as atropine [10]. However, long-term administration of these medications frequently indicates adverse reactions. For instance, levodopa and amantadine may induce gastrointestinal symptoms, such as nausea, vomiting, and loss of appetite, and in severe cases, may precipitate neuropsychiatric complications such as hallucinations and psychosis [11,12]. Although selegiline is generally well tolerated, its use carries potential risks of gastrointestinal ulcers or angina [13]. Prolonged treatment with benzhexol may result in cognitive impairment, dry mouth, dry eyes, and constipation [14,15] (Table 1 and Figure 1). Despite their symptomatic benefits, existing pharmacotherapies cannot effectively halt disease progression, let alone cure PD. Therefore, targeted immunotherapies against α-syn (such as monoclonal antibodies) have emerged as a major focus of current research. These therapies aim to enhance α-syn clearance and reduce its pathological accumulation within the brain, thereby improving cognitive and motor function [16,17]. In parallel, researchers have made remarkable progress in the development of non-α-syn-targeted therapeutic approaches over the past five years. These strategies encompass agents that modulate autophagy [18], counteract oxidative stress [19], combat mitochondrial dysfunction [20], suppress inflammatory responses [21], and confer neuroprotection [22], which, however, cannot be well translated into clinical practice. For example, monoclonal antibodies targeting α-syn exhibit low blood–brain barrier (BBB) permeability and can induce immune-related adverse events, i.e., amyloid-related imaging abnormalities. In Roche’s Phase II trial of prasinezumab for PD, 9% of subjects developed asymptomatic cerebral microbleeds (ARIA-H), with MRI revealing punctate hypointense signals in the basal ganglia region, likely reflecting antibody-activated microglia triggering perivascular inflammation [23]. Despite demonstrating neuroprotective effects in preclinical models, non-targeted therapies have yielded inconsistent results in clinical trials. Notably, creatine, intended to improve cellular energy metabolism, has failed to demonstrate disease progression delay in PD patients in large-scale long-term clinical trials [24], while the mitochondrial complex I activator ubiquinone (coenzyme Q10) was ineffective in delaying motor function deterioration [25].

Developing novel therapies capable of modifying the disease process of PD represents a significant challenge in neuropharmacology. In traditional medical practice, neuroactive compounds derived from natural sources (gastrodin and paeoniflorin, etc.) have long been used to ameliorate motor dysfunction. Their mechanisms of action primarily involve multi-target regulation of excitatory–inhibitory balance, suppression of abnormal neural signaling, and mitigation of neurotraumatic stress responses, thereby benefiting the restoration of functional homeostasis within the nervous system. Advances in modern pharmacology have not only provided scientific validation for these traditional applications but have also highlighted the neuroprotective and potential disease-modifying properties of individual compounds, including scorpion venom-derived peptides and paeoniflorin. Benefiting from their unique multi-target synergistic effects, relatively low incidence of adverse reactions, and well-defined neuroprotective actions, natural neuroactive compounds have emerged as a frontier focus in PD therapeutic research in recent years, fueling continued efforts to translate preclinical findings into clinical applications.

With the objective of assessing the therapeutic value of these neuroactive compounds in PD, we systematically searched PubMed, Web of Science, and CNKI databases spanning from 2010 to 2025, using combinations of keywords of Parkinson’s disease, neuroprotective herbal compounds, traditional medicine, multi-target mechanisms, natural product, autophagy, neuroinflammation, and oxidative stress. This study focuses on three key aspects: (1) clinical evidence supporting symptom management and disease-modifying effects; (2) molecular mechanisms underlying neuroprotective actions; (3) challenges encountered during the translation of preclinical research outcomes into clinical practice.

## 2. Natural Neuroactive Compounds in Clinical Anti-PD Effects

Patients with PD commonly experience age-related cognitive decline, often accompanied by sleep disturbances, anxiety, and motor complications. These symptoms typically worsen as age advances and tremor severity increases. Accumulating clinical evidence supports the applicability of Traditional Chinese Medicine (TCM) in addressing cognitive impairment and agitation in PD patients.

*Paeonia lactiflora* Pall. is an important natural source of neuroactive substances, containing multiple bioactive compounds with neuromodulatory and neuroprotective properties. White Peony Root Total Glycosides Capsules are formulated from these compounds extracted from *Paeonia lactiflora* Pall. In a study involving 61 PD patients, participants were randomized into a control group (*n* = 30) and a treatment group (*n* = 31), with the former group receiving standard PD treatment and the latter group receiving standard therapy supplemented with Paeonia Lactiflora Total Glycosides Capsules for six consecutive weeks. The two groups demonstrated remarkably lower Unified Parkinson’s Disease Rating Scale (UPDRS) scores relative to baseline; however, the treatment group exhibited a greater reduction (control group: 29.5 to 23.8; treatment group: 28.7 to 18.7). Additionally, the treatment group demonstrated more pronounced decreases in peripheral blood inflammatory markers of TNF-α, IL-6, and IL-1β, indicating favorable therapeutic outcomes [26]. Gastrodin, a natural neuroactive compound derived from *Gastrodia elata* Blume, a traditional Chinese medicinal herb, has demonstrated potent neuroprotective effects. In a randomized controlled clinical trial, 35 patients in the control group received levodopa monotherapy, while 35 patients in the treatment group were given gastrodin supplementation for 12 weeks in addition to their baseline levodopa regimen. Clinical outcomes were assessed using the Clinical Symptom Self-Assessment Scale. Both groups demonstrated improvement in clinical symptoms, but patients in the gastrodin treatment group exhibited significantly increased Montreal Cognitive Assessment (MoCA) scores, reflecting improved cognitive function, with particularly pronounced improvements in verbal fluency and delayed memory performance [27].

The aforementioned studies provide preliminary evidence supporting the clinical application of naturally derived neuroactive compounds, highlighting their potential to ameliorate motor and cognitive impairments in PD patients. However, these findings are constrained by small sample sizes (*n* ≤ 70), brief treatment durations (6–12 weeks), and the predominance of adjunctive therapy with conventional drugs, which complicates evaluation of their standalone efficacy. Therefore, while promising, they should be interpreted as preliminary and exploratory, underscoring the need for well-designed, large-scale, long-term, randomized, double-blind, placebo-controlled clinical trials to validate their independent therapeutic and disease-modifying effects. This situation also underscores the urgent need for in-depth investigations into their mechanisms of action. Only by elucidating how these compounds synergistically mitigate pathological processes at the molecular, cellular, and systemic levels can we lay a robust theoretical basis for the formulation of standalone natural botanical therapies in future research.

## 3. Mechanism of Action of Neuroactive Compounds from Natural Sources Against PD

**Figure 2:**
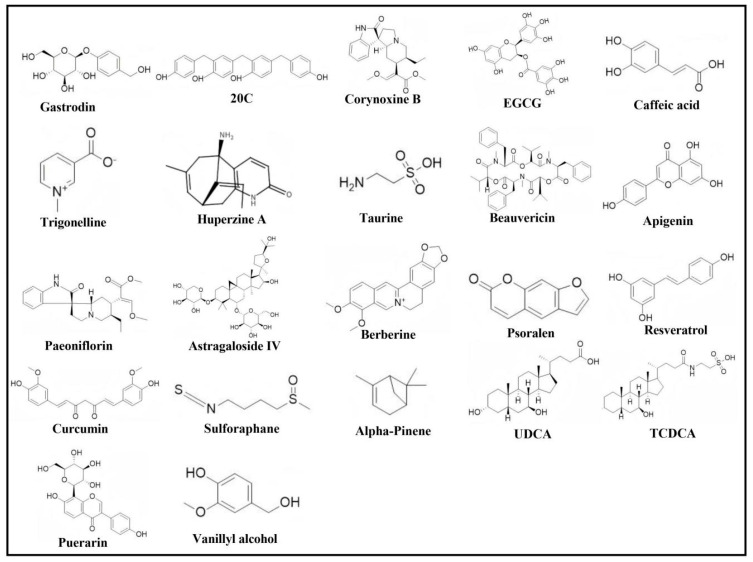
Chemical structures of major compounds.

Accumulating evidence indicates that neuroactive compounds from natural sources, including single-herb extracts, can intervene in the pathological progression of PD through multi-target mechanisms. These mechanisms primarily include inhibition of abnormal protein aggregation, such as α-syn, regulation of neurotransmitters, attenuation of neuroinflammation, suppression of oxidative stress, prevention of neuronal apoptosis, and restoration of mitochondrial dysfunction. The synergistic actions of these compounds theoretically assist in the development of multi-target PD treatment strategies for PD based on natural products. Table 2 lists the pharmacological actions and underlying mechanisms of selected neuroactive compounds, and Figure 2 illustrates the chemical structures of key compounds.

### 3.1. Anti-α-syn Pathological Deposits

**Figure 3:**
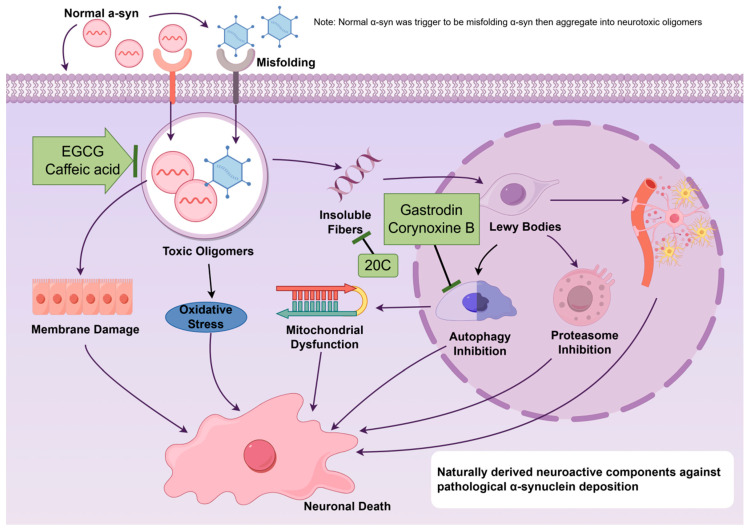
Neuroactive compounds targeting pathological α-syn deposition pathways (chemical structures of the compounds involved in this study, such as Gastrodin, 20C, Corynoxine B, EGCG, and Caffeic acid, are depicted in Figure 2).

The pathological accumulation of α-syn represents a central trigger in PD pathogenesis. Under genetic or environmental influences, soluble α-syn undergoes misfolding to form neurotoxic soluble oligomers [28]. These oligomers not only directly disrupt neuronal membrane integrity, leading to mitochondrial dysfunction and oxidative stress, but also aggregate into insoluble fibers, ultimately forming Lewy bodies within neurons [29]. This process itself directly impairs cellular function while simultaneously activating microglia to trigger chronic neuroinflammation. Concurrently, it inhibits two critical protein clearance pathways—the ubiquitin–proteasome system and the autophagy-lysosomal system—inducing accelerated accumulation of abnormal proteins. Ultimately, this cascade selectively drives the progressive loss of dopaminergic neurons in the substantia nigra, triggering the motor and non-motor symptoms characteristic of PD [30]. Furthermore, autophagy—a critical cellular pathway responsible for protein quality control and degradation—has been increasingly implicated in α-syn clearance, and its dysfunction has been consistently associated with toxic α-syn accumulation. Impaired autophagy, whether due to blocked autophagic flux or reduced lysosomal degradation efficiency, directly causes massive accumulation of toxic α-syn oligomers. These aggregates, in turn, further inhibit the ubiquitin–proteasome system and the autophagy-lysosomal pathway, ultimately exacerbating the collapse of the protein clearance machinery [31]. This section highlights natural compounds that act primarily on α-syn homeostasis, encompassing molecules that either directly inhibit α-syn misfolding and aggregation (e.g., 20C, EGCG, and caffeic acid) or promote its clearance via autophagy activation (e.g., gastrodin and corynoxine B) (Figure 3).

Gastrodin, a primary neuroactive compound derived from *Gastrodia elata* Blume, consists of an aglycone (4-hydroxybenzyl alcohol) linked to a β-D-glucopyranosyl moiety via an oxygen-containing glycosidic bond. This unique structure confers a characteristic hydrophilic–lipophilic balance and underlies its diverse biological activities [32]. 4-Hydroxybenzyl alcohol, a small-molecule aglycone, exhibits excellent BBB permeability. The phenolic hydroxyl group (-OH) on its benzene ring is capable of interacting with key autophagy proteins via hydrogen bonding while concurrently acting as an electron donor to scavenge reactive oxygen species (ROS), accordingly weakening oxidative stress damage to the autophagy-lysosomal pathway. The presence of β-glycosidic bonds markedly enhances gastrodin’s water solubility, but the resulting increase in molecular weight limits its free passage through the BBB. However, glycosidases in the gut microbiota can hydrolyze this bond, releasing the aglycone (i.e., gastrodin 4-hydroxybenzyl alcohol), which exerts direct neuroprotective effects [33,34]. In a rat model of PD where rats were administered 30 mg/kg MPTP for 7 consecutive days via intraperitoneal injection, daily intramuscular administration of gastrodin (0.2, 0.4, and 0.6 g/kg) for 14 consecutive days was found to significantly downregulate α-syn expression in the striatum [35]. Concurrently, expression of the microtubule-associated protein 1 light chain 3B (LC3B), an autophagy-related protein, was increased, indicating enhanced autophagic activity in striatal neurons, which may contribute to its neuroprotective effects [36]. In another cellular study, astrocytes underwent 30 min of pretreatment with 10 μM gastrodin before 24 h of exposure to lipopolysaccharide (LPS, 1 μg/mL) to establish a model of excessive autophagy. The results revealed that gastrodin pretreatment markedly inhibited both apoptosis and excessive autophagic activity, as evidenced by upregulation of the apoptosis-related protein B-cell lymphoma 2 (Bcl-2), downregulation of the pro-apoptotic protein Bcl-2-associated X (Bax), and activation of autophagy-related signaling pathways, including the PI3K/AKT/mTOR and p38 MAPK signaling pathways [37].

20C, another active compound derived from *Gastrodia elata* Blume, consists of two benzyl units connected together. Computational simulations indicate that 20C can bind into the hydrophobic pockets of α-syn fibrils, disrupting their β-sheet structure through a combination of hydrogen bonding and hydrophobic interactions, thereby inhibiting fibrillization. Studies have shown that 20C reduces the β-sheet content of α-syn by approximately 60%, leading to a notable reduction in neuronal inclusions [38]. In animal studies, a PD model was induced in mice via subcutaneous injection of 25 mg/kg MPTP. The regimen commenced on the third day of the experiment, with injections repeated every 4 days for a total of 10 doses. Concurrently, the mice received 20C (25, 50, or 100 mg/kg) for 53 consecutive days. Relative to the model group, 20C treatment reduced abnormal α-syn aggregation in the substantia nigra–striatal pathway and improved motor dysfunction [39].

Corynoxine B (Cory B) is an indole alkaloid from *Uncaria rhynchophylla* (Miq.) Miq. ex Havil., and directly binds to the C106 site of High mobility group box 1/2 (HMGB1/2) proteins via its oxazolidine structure, forming stable complexes via electrophilic interactions. This binding promotes the HMGB1/2-Beclin 1 interaction, enhances PI3K class III complex activity, initiates autophagosome formation, and exerts potent neuroprotective effects in neurological disorders. Corynoxine B (20 μmol/L) has been shown to cause autophagy and stimulate the clearance of α-syn in Neuro-2a (N2a) and PC12 mouse neuroblastoma cells [40]. In Prp-α-Syn A53T transgenic PD mouse models, Cory B was administered intraperitoneally as follows: 2-month-old mice received 20 mg/kg/d for one month; 10-month-old mice received 5, 10, and 20 mg/kg/d for one month; 15-month-old mice received 5 or 20 mg/kg every other day for two months. The results demonstrated that Cory B promotes autophagic clearance of α-syn by activating PI3K class III complex, enhancing LC3-II lipidation, and facilitating p62 degradation [41].

Epigallocatechin gallate (EGCG), the primary polyphenolic constituent of *Camellia sinensis* (L.) O. Ktze., has been shown to directly bind to α-syn monomers and redirect their assembly toward relatively inert, stable hollow spherical oligomers, thereby effectively preventing their fibrillization into toxic structures [42]. Notably, EGCG can even remodel mature fibrils already formed by α-syn, thereby reducing their toxicity [43]. In a study adopting a PD mouse model where neural damage was induced by a unilateral intrastriatal injection of pre-formed fibrils (PFFs, 1 μg/μL), pretreatment with EGCG at 10 mg/kg —delivered by daily intraperitoneal injection for 7 days prior to modeling and maintained for 6 consecutive months—exerted sustained neuroprotective effects. EGCG markedly alleviated anxiety-like behaviors and motor deficits in PD mice. Concurrently, it mitigated PFF-induced degeneration of tyrosine hydroxylase (TH)-immunopositive neurons and reduced the p-α-syn accumulation in the substantia nigra and striatum at the 6-month endpoint [44].

Caffeic acid, a phenolic neuroactive compound abundantly found in *Coffea arabica* Linn., possesses a catechol group that enables direct interaction with α-syn. This interaction effectively inhibits the formation of toxic oligomers by intervening with early oligomerization [45]. In transgenic PD mice expressing A53T mutant α-syn, oral administration of caffeic acid (10 mg/kg, once daily for 8 weeks) improved behavioral deficits, reduced dopaminergic neuron loss, enhanced autophagy, and decreased α-syn deposition in the substantia nigra [46].

Despite potent anti-α-syn deposition and pro-autophagy effects of these natural compounds in preclinical studies, they can hardly be translated into clinical application. Polyphenolic compounds such as EGCG and caffeic acid display low oral bioavailability, rapid metabolism, and limited BBB permeability [47]. Novel delivery strategies—including nanocarriers (e.g., liposomes and polymeric nanoparticles), prodrug design, and exploitation of BBB receptor-mediated transport—may represent promising research directions [48]. Research on the systemic toxicity, in vivo distribution, metabolic pathways, and long-term safety of compounds such as 20C and corynoxine B remains scarce. Standardized preclinical safety evaluations, along with optimized pharmacokinetics and dosage strategies, are therefore essential [49]. Moreover, most existing studies employ acute or subacute PD models, which inadequately reflect the chronic, progressive pathology of PD. All these findings necessitate the robust assessment of the disease-modifying potential of these compounds through more long-term intervention studies in chronic or transgenic models.

### 3.2. Regulation of Neurotransmitters

**Figure 4:**
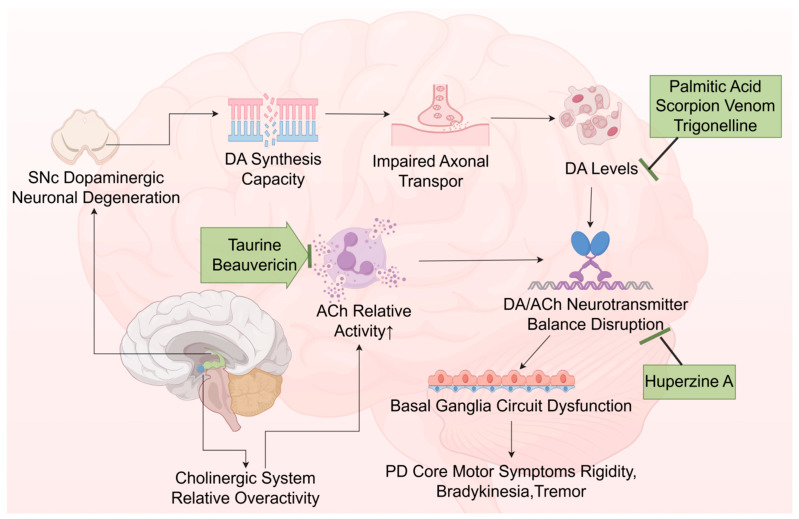
Regulatory role of neuroactive compounds in PD neurotransmitter dysregulation. The upward arrow (↑) indicates an increase in ACh relative activity. (chemical structures of the compounds involved in this study, such as Scorpion Venom, Palmitic acid, Trigonelline, Huperzine A, Taurine, and Beauvericin, are depicted in Figure 2).

Acetylcholine (ACh) and dopamine (DA) are key neurotransmitters widely distributed across the central and peripheral nervous systems, where DA exerts an inhibitory regulatory effect. Under physiological conditions, they maintain a dynamic balance with each other. The cell bodies of dopaminergic neurons in the SNc are the primary sites for DA synthesis. Degeneration or damage of SNc dopaminergic neurons markedly reduces their DA synthesis capacity, leading to insufficient axonal transport to striatal terminals and a consequent substantial decrease in striatal DA levels [50]. This reduction in dopaminergic neurotransmission leads to a relative increase in cholinergic system activity, thereby disrupting the normal neurotransmitter balance. This biochemical imbalance is considered a key pathophysiological mechanism underlying the core motor symptoms of PD (rigidity and bradykinesia, etc.) [51]. This section focuses on natural compounds that alleviate PD symptoms by modulating specific neurotransmitter systems, primarily including palmitic acid, scorpion venom, and trigonelline to increase DA levels; taurine and beauvericin to enhance Ach activity; and huperzine A to regulate the DA–Ach balance (Figure 4).

Scorpion Venom has been reported to alleviate motor disorders by reducing degenerative changes in striatal TH-positive neurons [52]. Simultaneously, it improves signal transmission efficiency in the nigrostriatal pathway by regulating dopamine release kinetics through its action on dopaminergic neuron receptors, such as D2 receptors [53]. Additionally, palmitic acid, a naturally occurring fatty acid commonly found in traditional Chinese herbs such as Scorpio, has been shown to regulate key neurochemicals, including α-syn, TH, DA, and serotonin (5-HT), thereby crucially mitigating the risk of neurodegenerative diseases, including PD [54].

Trigonelline is derived from natural plants such as *Trigonella foenum-graecum* L. [55,56]. In animal studies, a PD rat model was generated via unilateral striatal injection of 2.5 μg/μL 6-OHDA, administered one hour after the final intraperitoneal pretreatment with trigonelline (50 or 100 mg/kg, once daily for 3 consecutive days). The results revealed that trigonelline was associated with higher levels of DA, norepinephrine (NE), and 5-HT in the brains of model animals. This intervention not only improved motor function but also counteracted depression and anxiety commonly associated with PD [57].

Huperzine A, a natural alkaloid from the traditional Chinese medicinal plant *Lycopodium serratum* Thunb., functions as an acetylcholinesterase (AChE) inhibitor, thereby increasing synaptic ACh levels by reducing its degradation [58]. Its unique tetracyclic skeleton and specific functional groups collectively determine its highly efficient and selective inhibition of AChE. At physiological pH, the bridged primary amino group becomes protonated, allowing it to form cation-π interactions with aromatic amino acid residues at the base of the AChE active site—a key factor in its high activity. Meanwhile, the pyridone ring in the molecule can form hydrogen bonds with amino acids along the gorge edge, enhancing both binding selectivity and stability. The synergistic interaction of these structural features enables huperzine A to potently inhibit AChE, thereby stabilizing ACh and DA levels in the brain [59]. In a 6-OHDA-induced PD rat model (2 μg/μL), oral administration of 0.1 mg/kg huperzine A once daily for 4 weeks, starting 7 days after modeling establishment, was reported to improve spatial memory performance. Concurrently, huperzine A treatment increased DA levels and lowered AChE levels in PD rats’ brains [60].

Taurine, a sulfur-containing non-protein amino acid initially isolated from *Bovis Calculus*, can enhance GABAergic neurotransmission, strengthen learning and memory, and attenuate neural aging [61]. In a rotenone-induced PD rat model (1.5 mg/kg, intraperitoneally), concurrent oral administration of taurine at 5, 10, or 20 mg/kg for 28 days was associated with improved motor coordination, reduced motor deficits, and enhanced neuromuscular function [62]. It also increased AChE activity as well as levels of DA, glutathione (GSH), catalase (CAT), superoxide dismutase (SOD), and glutathione-S-transferase (GST) in the striatum and prefrontal cortex.

Beauvericin is the primary neuroactive compound derived from *Beauveria bassiana* and other traditional Chinese medicinal sources. In an in vitro PD model using SH-SY5Y cells that received 24 h of treatment with 6-OHDA (100 μmol/L), co-treatment with beauvericin (2.5, 5, and 10 μmol/L) significantly enhanced cell survival by up to 50% compared to the model group. It also inhibited acetylcholinesterase (AChE) activity and reduced neuronal apoptosis [63]. Further studies indicate that beauvericin exerts neuroprotective effects by modulating N-methyl-D-aspartate (NMDAR) receptors and inhibiting glutamate-mediated excitotoxicity.

Collectively, these compounds demonstrate promising potential for symptomatic management of PD by modulating neurotransmitter balance through multi-target mechanisms. However, several obstacles must be overcome before their translation into therapeutics. Huperzine A, a marketed AChE inhibitor, is limited in its long-term application for PD patients due to peripheral cholinergic side effects such as nausea and diarrhea [64]. Bioactive toxins, such as scorpion venom, although highly potent, present a narrow therapeutic window that demands stringent purification, quality control, and safety measures. For compounds such as trigonelline, taurine, and beauvericin, current research largely remains at the level of phenomenological descriptions and preliminary mechanistic studies, with limited data on dose–response relationships, pharmacokinetic (PK) studies, and potential drug–drug interactions. Future efforts should aim to reduce toxicity through structural modification while preserving activity, conduct systematic ADMET (absorption, distribution, metabolism, excretion, and toxicity) studies, and explore combination strategies with established PD therapeutics (e.g., levodopa) to evaluate potential synergistic effects and safety profiles.

### 3.3. Anti-Neuroinflammation and Antioxidant Stress

Regarding the complex pathogenesis of PD, neuroinflammation and oxidative stress do not operate in isolation; rather, they constitute a tightly interwoven and self-amplifying pathological network mediated by key signaling molecules, including NF-κB, NLRP3 inflammasome, and Nrf2 [65]. Oxidative stress generates abundant ROS, which activate NF-κB and trigger NLRP3 inflammasome assembly. Upon exposure to abnormal signals, microglia are activated and initiate a series of classical inflammatory signaling pathways, such as TLR4/NF-κB and NLRP3 inflammasome pathways. Activated microglia recognize abnormal signals through Toll-like receptors (TLRs), leading to NF-κB activation and the robust release of pro-inflammatory factors of tumor necrosis factor-α (TNF-α) and interleukin-1β (IL-1β), thereby establishing a chronic inflammatory microenvironment [66,67,68]. Under physiological conditions, NLRP3’s NACHT domain binds to its leucine-rich repeats (LRRs), maintaining the protein in a self-inhibited state. Exposure to pathogen-associated molecular patterns (PAMPs) or danger-associated molecular patterns (DAMPs)—such as endotoxins, viruses, or ATP—stimulates NLRP3 to be released from its autoinhibited conformation, exposing the NACHT domain and initiating oligomerization. The PYD domain at the N-terminus of NLRP3 recruits ASC adaptor proteins containing PYD domains, while the CARD domain of ASC takes charge of recruiting and activating pro-Caspase-1, completing inflammasome assembly [69]. Activated Caspase-1 further cleaves pro-IL-1β and pro-IL-18, promoting their maturation and inducing pyroptosis. It also cleaves gadermin D (GSDMD) and releases its N-terminal domain to form pores in the lipid bilayer, which stimulates mature IL-1β and IL-18 to be released from cells, thereby propagating downstream inflammatory responses [70]. Nuclear factor erythroid 2-related factor 2 (Nrf2) critically regulates cellular antioxidant responses, which, under physiological conditions, is sequestered by its inhibitory protein Keap1, promoting it to be ubiquitinated and degraded. Oxidative stress triggers the modification of cysteine residues in Keap1, stimulating Nrf2 to be released and subsequently translocated into the nucleus, where Nrf2 binds to the antioxidant response element (ARE), initiating the transcriptional expression of phase II detoxifying and antioxidant enzymes that include heme oxygenase-1 (HO-1), NAD(P)H:quinone oxidoreductase 1 (NQO1), and glutathione S-transferase (GST), thereby mediating oxidative stress responses. This upstream inhibition suppresses the NF-κB and NLRP3 pathway activation [71,72]. Notably, Nrf2 activity within the cell nucleus is tightly regulated. After completing its transcriptional activation function, it is promptly cleared through Keap1-dependent mechanisms—such as being transported back to the cytoplasm or binding to nuclear degradation complexes—to ensure the moderation and terminability of antioxidant responses, thereby maintaining the dynamic equilibrium of redox homeostasis [73]. This interconnected network underscores the multi-targeted pathology of PD, providing a rationale for the broad neuroprotective efficacy of naturally occurring bioactive compounds such as berberine, curcumin, and sulforaphane. These compounds act synergistically on multiple critical nodes within this network, thereby more effectively disrupting pathogenic feedback loops and restoring intracellular homeostasis.

#### 3.3.1. Anti-Neuroinflammation

**Figure 5:**
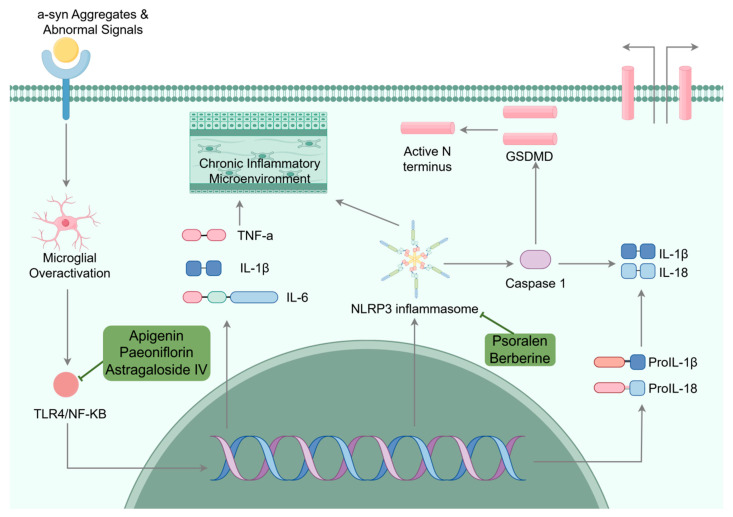
Mechanism of neuroinflammatory Suppression by neuroactive compounds (chemical structures of the compounds involved in this study, such as Apigenin, Paeoniflorin, Astragaloside IV, Berberine, Psoralen, are depicted in Figure 2).

This section focuses on natural compounds whose primary mechanism of action is the modulation of neuroinflammation. These include compounds that inhibit the NF-κB pathway, such as apigenin, paeoniflorin, and astragaloside IV, as well as compounds that inhibit NLRP3 inflammasome activation, including berberine and psoralen (Figure 5).

##### NF-κB Signaling Pathway

Multiple natural bioactive compounds can inhibit the NF-κB pathway. Flavonoids, such as apigenin, can downregulate TNF-α expression through suppressing NF-κB signaling [74]. In a study using the rat mesencephalic neuron × neuroblastoma hybrid cell line MES23.5 (MES23.5 cells), pretreatment with 10 μmol/L apigenin for 2 h restricted the subsequent activation of the NF-κB pathway and the release of inflammatory mediators induced by 200 μmol/L MPP^+^ (24 h). This mechanism underlies the fact that apigenin protects against dopaminergic neurons [75].

*Paeonia lactiflora* Pall., the dried root of a plant in the *Ranunculaceae* family, has been used as a TCM for over two thousand years. Its primary bioactive constituent, paeoniflorin, consists of a glucose moiety linked to a pinane monoterpene skeleton via a β-glycosidic bond. The glucose moiety enhances water solubility but limits BBB permeability, whereas the hydrophobic pinane skeleton can embed into the hydrophobic pockets of inflammatory proteins such as TLR4, thereby blocking protein dimerization [76,77]. Paeoniflorin exhibits anti-inflammatory properties, as evidenced by its efficacy in inhibiting the TLR4/NF-κB pathway and reducing the production of proinflammatory cytokines of IL-17, IL-6, and TNF-α [78]. In BV-2 microglial cells, 1 h of pretreatment with paeoniflorin (10–200 μM) prior to LPS exposure (200 ng/mL) was associated with a dose-dependent enhancement in cell viability and reduction in nitric oxide (NO) release. Additionally, paeoniflorin led to the downregulation of the protein levels of Inducible nitric oxide synthase (iNOS) and Cyclooxygenase-2 (COX-2), prevented NF-κB nuclear translocation by inhibiting phosphorylation of its inhibitory protein IκB-α, and subsequently restricted the MAPK pathway activation, attributed to the attenuated phosphorylation of p38, Erk, and Jnk. In vivo, mice were orally administered paeoniflorin (10, 20 mg/kg) daily for 3 consecutive weeks, with daily intraperitoneal LPS injections (0.25 mg/kg) administered during the final week (days 15–21) to induce the model. The results revealed that Paeoniflorin significantly alleviated LPS-induced inflammatory injury, enhanced spatial learning and memory, improved cognitive function, mitigated memory deficits, suppressed reactive ROS production, and downregulated nuclear NF-κB p65, iNOS, and COX-2 expression [79].

Astragaloside IV (AS-IV), a primary bioactive compound derived from *Astragalus membranaceus* (Fisch.) Bunge, contains glycosyl groups (e.g., glucuronic acid) that enhance its water solubility, participate in the recognition of TLRs on immune cell surfaces, regulate microglial polarization toward the M2 anti-inflammatory phenotype, and alleviate neuroinflammation [80]. In an acute PD mouse model induced by MPTP (18 mg/kg, administered via intraperitoneal injection four times at 2-h intervals), mice were given AS-IV (10 mg/kg, 40 mg/kg) by oral gavage over a 14-day regimen. The treatment commenced 7 days prior to MPTP injection (twice daily) and continued for 7 days thereafter. The results indicated that AS-IV ameliorated motor deficits in PD mice, accompanied by elevated TH expression and microglial polarization shift from the pro-inflammatory M1 to the anti-inflammatory M2 phenotype [81]. In terms of mechanism, AS-IV weakened the expression and phosphorylation of key signaling molecules, including TLR4, MyD88, p-p38, p-JNK, and p-NF-κB, which consequently reduced pro-inflammatory cytokines (IL-1β, IL-6, TNF-α) and elevated anti-inflammatory factors (IL-4, IL-10). Validation experiments employed the specific TLR4 agonist LPS-EB Ultrapure, selectively activating the TLR4/NF-κB pathway and elevating the mRNA levels of TLR4, NF-κB, IL-1β, IL-6, and TNF-α. Collectively, AS-IV exhibits anti-neuroinflammatory activities through the inhibition of this pathway [82].

##### NLRP3 Inflammasome Pathway

The NLRP3 inflammasome activation serves as a pivotal bridge linking misfolded proteins in PD, mitochondrial stress, and chronic neuroinflammation, ultimately contributing to neuronal death. These findings collectively position the NLRP3 inflammasome as a highly promising therapeutic target [70].

Berberine, a common isoquinoline alkaloid extracted from traditional Chinese medicinal plants, *Coptis chinensis* Franch. and *Phellodendron amurense* Rupr., has been reported to target the NLRP3 inflammasome by inhibiting its assembly and activation. This is associated with downregulation of downstream effectors, including caspase-1 and GSDMD, leading to decreased secretion of mature pro-inflammatory cytokines (IL-1β and IL-18), and mitigation of neuroinflammation [83,84]. In MPTP-induced PD mouse models (30 mg/kg/d, subcutaneous injection for five consecutive days), berberine (BBR) (50 mg/kg) was administered via oral gavage starting 7 days prior to modeling and continued once daily for 21 days. The results revealed that BBR intervention not only significantly alleviated behavioral impairments in PD mice but also effectively reduced neurotoxicity in the substantia nigra, and inhibited NLRP3 inflammasome activation—evidenced by downregulated expression of key molecules NLRP3, ASC (PYCARD), cleaved caspase-1, and mature IL-1β, along with enhanced autophagy activity in this brain region [85].

Psoralen is a bioactive compound derived from the traditional Chinese medicinal plant *Psoralea corylifolia* Linn. In a PD model co-cultured with microglia and astrocytes, induced by LPS (100 ng/mL, 5.5 h) combined with ATP (5 mM, 30 min), pretreatment with psoralen (1 μM, 1 h) facilitated direct binding between the N-terminal and L-type receptor domains of NLRP3, thereby blocking serine phosphorylation at position 658. This intervention inhibited NLRP3 inflammasome activity in microglia and astrocytes, lowered the expression of inflammatory mediators, including IL-1β, IL-6, and TNF-α, meliorated PD-like symptoms, and mitigated dopaminergic neuronal loss. These protective effects were further corroborated in in vivo PD models [86].

The therapeutic strategy targeting the NF-κB and NLRP3 inflammasome pathways demonstrates great potential in the anti-inflammatory treatment for PD. However, neuroinflammation is a complex and dynamic process, and prolonged or complete suppression of key inflammatory pathways, like NF-κB, may disrupt normal immune surveillance and increase susceptibility to infection. To address this, future research should prioritize modulating rather than completely inhibiting inflammatory signaling. One potential strategy is to stimulate microglial polarization toward the M2 phenotype, as previously demonstrated by Astragaloside IV. Concurrently, their long-term efficacy and safety should be assessed in chronic neuroinflammatory models that better replicate human pathology, such as humanized mouse models or brain organoids.

#### 3.3.2. Antioxidant Stress

**Figure 6:**
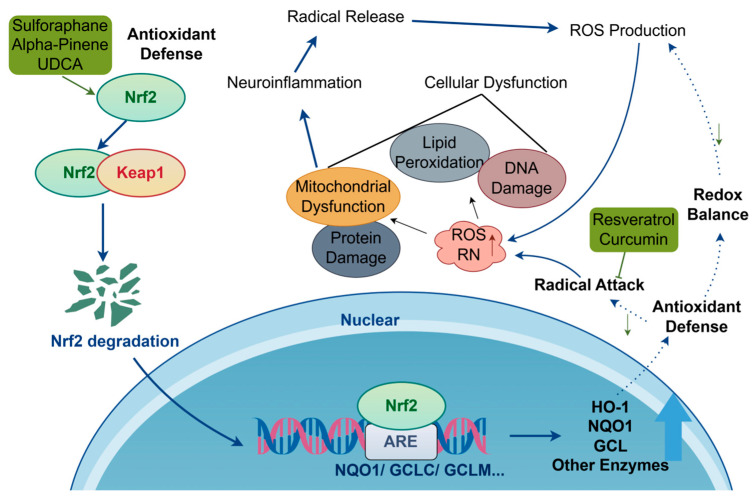
Antioxidant stress response pathway of neuroactive compounds. The upward arrow (↑) denotes overproduction of ROS and RNS. The downward arrow (↓) indicates enhanced inhibition and a reduction in ROS generation due to improved redox homeostasis following the augmentation of the antioxidant defense system (chemical structures of the compounds involved in this study, such as Resveratrol, Curcumin, Sulforaphane, Alpha-pinene, UDCA, are depicted in Figure 2).

This section addresses natural compounds that exert neuroprotective effects primarily by mitigating oxidative stress. These include direct radical scavenging (e.g., resveratrol and curcumin) and compounds that activate the Nrf2 signaling pathway (e.g., sulforaphane, alpha-pinene, and UDCA) (Figure 6).

##### Direct Radical Scavenging

Oxidative stress in PD involves an imbalance between the excessively produced ROS and reactive nitrogen species (RNS) and the cellular antioxidant defense system. Accumulation of these free radicals attacks and damages all macromolecules within the cell, including lipids, proteins, DNA, and mitochondria, ultimately contributing to the dysfunction and death of dopaminergic neurons [87]. Numerous neuroactive compounds of natural origin mitigate oxidative damage by directly scavenging free radicals and interrupting radical chain reactions, thereby stabilizing redox homeostasis and protecting neurons.

Resveratrol, a polyphenol belonging to the quinone class, is widely present in medicinal herbs such as *Reynoutria japonica* Houtt [88]. Its unique styrene-diphenyl structure exerts direct antioxidant effects by providing electrons to neutralize free radicals. Additionally, this structure enables it to function as a signaling molecule that specifically activates the SIRT1 deacetylase enzyme, which enhances cellular stress resistance similarly to caloric restriction [89]. In α-synuclein transgenic Drosophila PD models, 21-day resveratrol (15, 30, or 60 mg/kg) treatment was associated with attenuation of cognitive impairment and oxidative damage. This intervention enhanced CAT activity while reducing NO and malondialdehyde (MDA) levels, thereby mitigating neuropathologies induced by oxidative stress [90].

Curcumin, a bioactive compound extracted from the rhizomes of *Curcuma longa* L., possesses a β-diketone structure that confers potent free radical scavenging capability. Additionally, it inhibits the JNK/p38 MAPK pathway, thereby reducing rotenone-induced apoptosis rates [91]. In a rotenone-induced PD rat model (2.5 mg/kg), continuous oral administration of 200 mg/kg curcumin for 5 weeks resulted in lower MDA, NO, and AChE levels, alongside higher GSH, SOD, CAT, and DA levels, indicating both significant free radical scavenging and substantial neuroprotective potential [92]. However, curcumin’s low bioavailability severely limits its clinical application; hence, efforts are made to develop novel delivery systems (nanocarriers and liposomes, etc.) to strengthen its brain exposure.

##### Nrf2/ARE Pathway

Nrf2 is a central regulator of cellular antioxidant defenses, and many naturally occurring neuroactive compounds exert their effects by mediating this pathway.

Sulforaphane (SFN), an isothiocyanate compound, is recognized as one of the most potent natural Nrf2 inducers. It activates Nrf2 by alkylating cysteine residues of Keap1 [93]. In a 6-OHDA-induced PD model, oral administration of SFN (5 mg/kg, twice weekly for 4 weeks) following model induction exerted neuroprotective effects. SFN not only enhanced antioxidant capacity via the Nrf2/ARE pathway but also alleviated glial cell-mediated neuroinflammation and promoted α-syn clearance [94].

Alpha-pinene, a monoterpene compound widely present in plant essential oils, has been reported to enhance Nrf2 stability and transcriptional activity through multiple mechanisms [95,96]. In a PC12 cell model of H_2_O_2_-induced oxidative stress (0.1 mM, 30 min), pretreatment with α-pinene (10 or 25 μM) for 24 h was associated with significant upregulation of critical antioxidant enzymes, including SOD, glutathione reductase (GR), CAT, glutathione peroxidase (GPx), and HO-1. Furthermore, α-pinene reduced caspase-3 activity, enhanced ROS scavenging, and induced the nuclear factor Nrf2. These findings ascertain a valuable therapeutic role of α-pinene in restoring oxidative balance in PD [97].

Ursodeoxycholic acid (UDCA) is a chenodeoxycholic acid compound with significant antioxidant activity. In a PD mouse model, pretreatment with UDCA (50 mg/kg for 3 days) before intraperitoneal injection of MPTP (40 mg/kg) resulted in upregulation of key components of the antioxidant defense system, including Nrf2, its stabilizer DJ-1, as well as effector enzymes of HO-1 and GPx. Furthermore, in an SH-SY5Y cell model of PD induced by MPP^+^ (1 mM), pretreatment with 100 µM UDCA for 12 h alleviated MPP^+^-induced oxidative stress through Nrf2 pathway activation [98]. According to consistent evidence from both in vitro and in vivo studies, UDCA potentially serves as a therapeutic agent for PD, with its mechanism of action closely linked to the Nrf2 pathway-mediated antioxidant stress.

Direct free radical scavenging and Nrf2/ARE signaling activation represent key strategies for counteracting oxidative stress in PD. Nonetheless, clinical translation remains challenging. For instance, curcumin exhibits low bioavailability, similar to EGCG, while resveratrol undergoes rapid metabolism in humans, resulting in brain concentrations far below those effective in animal studies. This highlights the translational gap from species differences and dose conversion. For compounds like sulforaphane and alpha-pinene, the optimal doses, therapeutic windows, and potential long-term effects on Nrf2 pathway homeostasis remain unclear [99]. In summary, future research should move beyond the mere discovery of novel antioxidants. Greater emphasis should be placed on structural optimization through medicinal chemistry, enhancement of PK/PD properties, development of smart and efficient brain-targeted delivery systems, and execution of early-stage clinical studies. These efforts are critical to validate the efficacy signals identified in preclinical models and to establish safe and effective dosing regimens for human use.

### 3.4. Anti-Mitochondrial Dysfunction

**Figure 7:**
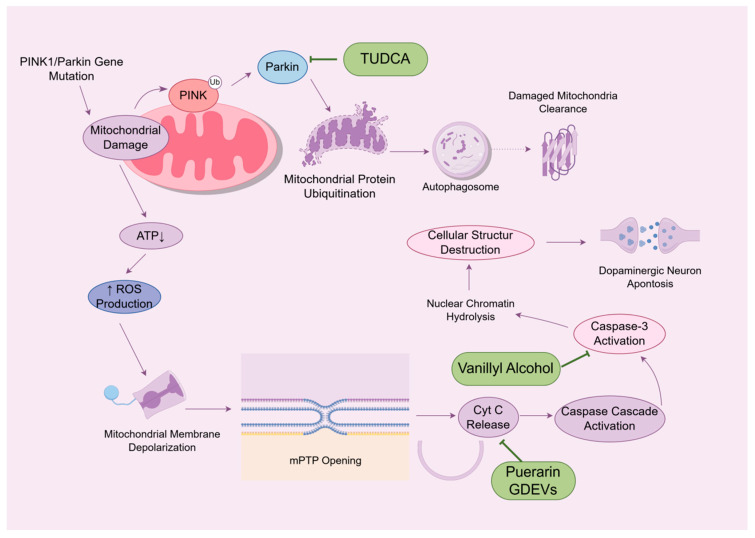
Neuroactive compounds’ effects against mitochondrial dysfunction. The upward arrow (↑) indicates a large production of ROS. The downward arrow (↓) denotes a decline in ATP synthesis capacity (chemical structures of the compounds involved in this study, such as TUDCA, Puerarin, GDEVs, Vanillyl alcohol, are depicted in Figure 2).

In PD, mitochondrial dysfunction serves as the core initiating factor. Specifically, pathogenic factors (such as PINK1/Parkin gene mutations or environmental toxins) compromise mitochondrial integrity and function while simultaneously impairing the PINK1/Parkin-dependent mitochondrial autophagy mechanism. This disruption prevents the timely clearance of dysfunctional mitochondria, leading to their excessive accumulation. This results in reduced ATP generation, massive ROS production, and heightened mitochondrial outer membrane permeability—a key event primarily mediated by oligomerization of pro-apoptotic proteins Bax/Bak, forming pores that release apoptotic factors such as cytochrome c (Cyt c) from the intermembrane space into the cytoplasm. Cyt c then directly binds apoptotic protease-activating factor 1 (Apaf-1) and assembles into a complex known as the ‘apoptosome’ with deoxyadenosine triphosphate (dATP), accordingly recruiting and activating the initiator caspase-9. The activated caspase-9 works on cleaving and activating the downstream effector caspase-3, thereby executing apoptosis and contributing to selective dopaminergic neuron loss [100,101]. This section focuses on natural compounds that primarily target mitochondrial dysfunction, including modulation of mitochondrial autophagy (e.g., TUDCA) and regulation of mitochondrial apoptosis (e.g., puerarin and GDEVs) (Figure 7).

#### 3.4.1. Regulation of Mitochondrial Autophagy System Imbalance

As a selective form of autophagy, mitophagy functions as a vital quality control mechanism by selectively targeting impaired mitochondria for removal, which is integral to sustaining energy homeostasis and promoting neuronal survival. Its core regulatory pathway involves the PINK1/Parkin pathway. In the case of mitochondrial damage, PINK1 accumulates stably on the outer mitochondrial membrane and becomes activated, subsequently activating Parkin. After the activation of Parkin, mitochondrial outer membrane proteins are ubiquitinated, ultimately guiding the engulfment and degradation of damaged mitochondria by autophagosomes [102,103].

Taurocholic acid (TUDCA) is a hydrophilic bile acid whose anti-PD effects are closely associated with stabilizing mitochondrial function and activating PINK1/Parkin-mediated mitochondrial autophagy. In an MPTP-induced mouse PD model (30 mg/kg, intraperitoneal injection for five consecutive days), TUDCA was administered 50, 100, or 200 mg/kg once daily for 10 days via intraperitoneal injection. The results revealed that TUDCA markedly improved motor deficits in mice by upregulating the PINK1/Parkin pathway, enhancing mitochondrial autophagy, facilitating the clearance of damaged mitochondria, improving cellular energy metabolism, and protecting dopaminergic neurons. These mechanisms were further corroborated by in vitro studies [104].

#### 3.4.2. Mitochondrial Apoptosis Regulation

Mitochondria, the primary organelles responsible for cellular energy production, exhibit altered membrane permeability in PD patients‘ substantia nigra. Persistent mitochondrial stress signals, including ROS accumulation and calcium overload, promote abnormal opening of the mitochondrial permeability transition pore (mPTP) and activate pro-apoptotic Bcl-2 family members (e.g., Bax). These events facilitate the release of Cyt c and other pro-apoptotic factors from the mitochondrial intermembrane space into the cytoplasm. Subsequently, Cyt c directly binds to proteins such as Apaf-1 to form the apoptosome, accordingly activating the caspase protease cascade. Activated caspases hydrolyze nuclear chromatin and other critical cellular proteins, disrupting the overall cellular structure and function, ultimately resulting in apoptosis [105].

Puerarin, an isoflavone compound from *Pueraria lobata* (Willd.) Ohwi, possesses an 8-C-glycosylated 7,4’-dihydroxyisoflavone core. Its phenolic hydroxyl group directly scavenges ROS and preserves mitochondrial membrane potential, thus inhibiting mPTP opening and mitochondrial apoptosis. Additionally, the glycosidic structure enhances water solubility and bioavailability, facilitating BBB penetration for therapeutic action [106]. In a mouse model of subarachnoid hemorrhage (SAH) established via intravascular puncture, a single intraperitoneal administration of puerarin (100 mg/kg) 2 h prior to surgery dose-dependently induced Bcl-2 expression upregulation, Bax expression downregulation, and Bcl-2/Bax ratio elevation. This shift effectively suppressed Bax-mediated mitochondrial membrane permeabilization, thereby blocking cytochrome c release, apoptosome formation, and caspase activation, ultimately inhibiting mitochondrial apoptosis [107].

Gardenia-derived extracellular vesicles (GDEVs), isolated from the fruit of *Gardenia jasminoides* J. Ellis, are rich in bioactive substances and have been well studied in recent years. In a rotenone-induced (1 μM, 48 h) PC12 cell PD model, pretreatment with GDEVs (20–50 μg/mL) for 4 h markedly mitigated mitochondrial membrane potential depolarization. The underlying mechanism involves the modulation of p38 MAPK and p53 phosphorylation within the MAPK/p53 signaling pathway, which subsequently elevated the Bcl-2/Bax ratio, ultimately inhibiting Bax/Bak-mediated Cyt c release and the downstream caspase-3 activation [108].

### 3.5. Anti-Neurological Cell Apoptosis

Apoptosis is an active, genetically programmed process of cell death triggered by a cascade of specific signals. While physiological neuronal apoptosis normally occurs to some extent during development and aging, this process is pathologically amplified in PD, contributing to the specific loss of dopaminergic neurons in the substantia nigra pars compacta [109]. According to research findings, PD patients exhibit a marked increase in caspase-3-positive neurons in the substantia nigra, with caspase-3 activation detectable even prior to classic apoptotic morphological features. Furthermore, its spatial distribution closely parallels regions of neuronal loss, highlighting caspase-3’s pivotal role as the core executor in neuronal apoptosis within PD [110]. As shown in Figure 7, caspase-3 activation constitutes the terminal execution step of the mitochondrial apoptosis pathway (cytochrome c release → apoptosome formation → caspase-9 activation) and serves as a common downstream convergence point for multiple apoptotic signals.

Vanillyl alcohol is a neuroactive component derived from *Gastrodia elata* Blume. The para-aldehyde moiety on the vanillin phenyl ring forms a covalent bond with Cys106 of HMGB1, thereby blocking HMGB1/TLR4 interaction, suppressing NF-κB nuclear translocation, and ultimately reducing neuronal apoptosis [111]. In an in vitro PD model established using MN9D mouse dopaminergic neurons treated with MPP^+^, pretreatment with 10 or 20 μM vanillin for 4 h prior to 48 h exposure to 25 μM MPP^+^ led to a lower Bax/Bcl-2 ratio and correspondingly decreased apoptosis [112]. Additionally, in a PD rat model induced by intraperitoneal injection of rotenone (2.5 mg/kg for 45 days), oral administration of vanillin (20 mg/kg for 45 consecutive days) contributed to improvements in motor dysfunction, coupled with higher Bcl-2 expression, lower Bax expression, elevated Cyt c expression, and expression downregulation of caspase-3, caspase-8, and caspase-9 [113]. Taken together, vanillyl alcohol exerts multi-targeted actions that converge on caspase-3 inhibition, thereby suppressing neuronal apoptosis, preserving neuronal structure and function, and significantly improving motor dysfunction in PD rats.

**Table 2:** Research on the mechanisms of anti-PD effects of major neuroactive compounds from natural sources.

Active Compounds	Source	Model	Treatment	Major Finding	Ref.
Gastrodin	*Gastrodia elata* Blume.	MPTP 30 mg/kg, i.p., for 7 days consecutively, PD rat model	0.2, 0.4, 0.6 g/kg doses of gastrodin, i.m., 14 days	α-syn ↓ LC3-II↓ Autophagy activity ↑	[35,36]
		LPS 1 μg/mL, inducing astrocytes for 24 h, excessive autophagy model	10 μM pretreatment for 30 min	Bcl-2 ↑ Bax ↓ P62↓ PI3K ↓ mTOR ↓ p38 MAPK ↑	[37]
20C		SH-SY5Y and H4 Cell Models Overexpressing SNCA (A53T)	10 μM treatment for 24 h	α-syn ↓ Disrupt its β-sheet structure α-syn inclusion formation ↓	[38]
		MPTP 25 mg/kg, i.d., 4 days per course, 10 courses total, PD mouse model	25, 50, 100 mg/kg, i.g., administered starting 3 days prior to modeling, for 53 consecutive days.	α-syn ↓ Improve motor dysfunction	[39]
Corynoxine B	*Uncaria rhynchophylla* (Miq.) Miq. ex Havil	20 μmol/L Cory induces autophagy in N2a and PC12 cells for 6–12 h.	20 μmol/L, 6–12 h	A53T α-syn ↓ p-Akt ↓ p-mTOR ↓ p-p70 S6 Kinase ↓	[40]
		Prp-α-Syn A53T transgenic PD mouse model	2-month-old mice: 20 mg/kg daily for 1 month; 10-month-old mice: 5, 10, or 20 mg/kg daily for 1 month; 15-month-old mice: 5 mg/kg or 20 mg/kg administered every other day for 2 months, i.g.	PI3KC3 ↑ LC3-II ↑ p62 ↓ α-syn ↓	[41]
EGCG	*Camellia sinensis* (L.) O. Ktze.	Single injection of 1 μg/μL PFFs (3 μL) into the striatal region of the left hemisphere in PD mouse models	10 mg/kg, i.p., begin administration 7 days prior to modeling and continue for 6 consecutive months.	TH^+^ ↑ p-α-syn ↓	[44]
Caffeic acid	*Coffea arabica* Linn.	A53T α-syn Transgenic PD Mouse Model	10mg/kg, i.g., once daily for 8 consecutive weeks	DA ↑ α-syn ↓	[46]
Trigonelline	*Trigonella foenum-graecum* L.	Unilateral striatal injection of 2.5 μg/μL 6-OHDA, 5 μL, PD rat model	50, 100 mg/kg doses administered preoperatively, i.g., once daily for 3 consecutive days, until 1 h prior to surgery.	DA ↑ NE ↑ 5-HT ↑	[57]
Huperzine A	*Lycopodium serratum* Thunb	Single injection of 2 μg/μL 6-OHDA into the ventral tegmental area and substantia nigra pars compacta of the right midbrain, PD rat model	0.1 mg/kg, i.g., once daily for 4 consecutive weeks	DA ↑ AchE ↓	[60]
Taurine	*Bovis Calculus*	1.5 mg/kg rotenone, i.g., administered orally every other day for 14 days, PD rat model	5, 10, 20 mg/kg, i.g., once daily for 28 consecutive days	Ach ↓ GSH ↓ CAT ↓ SOD ↓ GST ↓	[62]
Beauvericin	*Beauveria bassiana*	100 μmol/mL 6-OHDA-induced SH-SY5Y cells for 24 h, PD cell model	2.5, 5, 10 mg/mL treatment, incubate at 37 °C for 10 min	AchE ↓ NMDAR ↑ Excitotoxicity ↓	[63]
Apigenin	*Apium graueolens* L.	200 μmol/L MPP^+^-induced MES23.5 cells for 24 h, PD cell model	10 μmol/L, pretreated for 2 h	TNF-α ↓ NF-κB ↓	[75]
Paeoniflorin	*Paeonia lactiflora* Pall.	200 ng/mL LPS-induced BV2 cells for 24 h	10, 20, 50, 100, 200 μM pretreated for 1 h followed by co-incubation for 24 h.	ROS ↓ IκB-α ↓ NF-κB ↓ IRF3 ↑ p65 ↓ iNOS ↓ COX-2 ↓	[79]
		LPS, i.p., 0.25 mg/kg/d, for 7 consecutive days, in a mouse model of neuroinflammation and memory impairment	10, 20 mg/kg, i.g., once daily, for 3 consecutive weeks		
Astragaloside IV	*Astragalus membranaceus* (Fisch.) Bunge.	MPTP, 18 mg/kg, i.p., 4 times, every 2 h, PD mouse model	10 mg/kg, 40 mg/kg, i.g., twice daily, starting 7 days prior to MPTP injection and continuing for 7 days after MPTP injection.	TLR4 ↓ MyD88 ↓ p-p38 ↓ p-JNK ↓ p-NF-κB ↓ IL-1β ↓ IL-6 ↓ TNF-α ↓ IL-4 ↑ IL-10 ↑	[81,82]
Berberine	*Coptis chinensis* Franch., *Phellodendron amurense* Rupr.	MPTP, 30 mg/kg/d, s.c, 5 consecutive days, PD mouse model	50 mg/kg berberine pretreated for 7 days, once daily, and continued for 21 days after MPTP injection.	NLRP3 ↓ PYCARD ↓ CASP1 ↓ IL-1β ↓	[85]
Psoralen	*Psoralea corylifolia* Linn.	LPS (100 ng/mL, 5.5 h) combined with ATP (5 mM, 30 min) induced PD model in co-cultured microglia and astrocytes	1 μM pretreatment for 1 h	NLRP3 ↓ IL-1β ↓ IL-6 ↓ TNF-α	[86]
Resveratrol	*Reynoutria japonica* Houtt.	α-syn Transgenic Drosophila PD Model	15, 30, 60 mg/kg, administered as dietary supplements, 21 d	CAT ↑ NO ↓ MDA ↓	[90]
Curcumin	*Curcuma longa* L.	2.5 mg/kg rotenone, i.g., 5 weeks, PD rat model	200 mg/kg, i.g., begin pretreatment 5 days prior to modeling, followed by co-administration with rotenone for 5 weeks.	MDA ↓ NO ↓ AchE ↓ GSH ↑ SOD ↑ CAT ↑ DA ↑	[92]
SFN	*Brassica oleracea* L. *var. italica* Plenck	Stereotaxic single injection of 4 μg/μL 6-OHDA into the left striatum PD rat model	5 mg/kg, i.g., begin 1 h after molding, twice weekly for 4 weeks.	ERK ↓ Nrf2 ↑ GSH ↑ GR ↑ GST ↑ α-syn ↓	[94]
Alpha-Pinene	Plant essential oils	0.1 mM H_2_O_2_, 30 min, PC12 Cell Oxidative Stress Injury Model	10 μM and 25 μM, pretreated for 24 h	CAT ↑ SOD ↑ GPx ↑ GR ↑ HO-1 ↑ Capase-3 ↓ ROS ↓ Nrf2 ↑	[97]
UDCA	*Cow bezoar*	A single dose of 40 mg/kg MPTP administered i.p. PD mouse models	50 mg/kg once daily for 3 consecutive days of premedication	Nrf2 ↑ DJ-1 ↑ HO-1 ↑ GPx ↑	[98]
		SH-SY5Y cells treated with 1 mM MPP^+^ for 24 h	100 μM, pretreated for 12 h		
TCDCA	Bile acid	30 mg/kg MPTP, i.p., 5 consecutive days, PD mouse model	50, 100, 200 mg/kg/d, i.g., once daily for 10 consecutive days	Pink1 ↑ Parkin ↑	[104]
		200 ng/mL LPS-stimulated BV2 microglia for 24 h	25, 50, and 100 μM TCDCA treatment for 5 h		
Puerarin	*Pueraria lobata* (Willd.) Ohwi	Intravascular Puncture Method for Establishing a Mouse Subarachnoid Hemorrhage (SAH) Model	Pretreatment with a single dose of 100 mg/kg puerarin administered 2 h prior to surgery.	ROS ↓ Bcl-2 ↑ Bax ↓ Bcl-2/Bax ↑	[107]
GDEVs	*Gardenia jasminoides* J.Ellis	1 μM rotenone, treated PC12 cells for 48 h	20–50 μg/mL pretreatment for 4 h	p38 MAPK ↓ p53 ↓ Bcl-2/Bax ↑ Caspase-3 ↓	[108]
Vanillyl alcohol	*Gastrodia elata* Blume.	25 μM MPP^+^, treated MN9D cells for 48 h	10, 20 μM, pretreated for 4 h	Bax/Bcl-2 ↓ Apoptosis ↓	[112]
		2.5 mg/kg rotenone, i.g., for 45 consecutive days, PD rat model	20 mg/kg, i.g., 45 consecutive days	Bcl-2 ↑ Bax ↓ Cyt c ↑ Caspase-3,8,9 ↓	[113]

Note: Arrow symbols indicate significant changes in the indicators after drug administration: an upward arrow (↑) denotes up-regulation or enhanced activity; a downward arrow (↓) denotes down-regulation or reduced activity.

## 4. Conclusions and Outlook

The pathophysiological complexity of PD stems from the multilayered and dynamic interactions between various pathogenic factors, including genetic predisposition and environmental toxicant exposure, and pathological mechanisms such as abnormal α-syn aggregation, neuroinflammatory activation, and oxidative stress. These factors collectively drive dopaminergic neuron degeneration in the substantia nigra through a cascade of neuronal-glial-immune system interactions [114,115]. Although PD is traditionally associated with risk factors such as neurological damage and cerebrovascular disorders, emerging evidence indicates that aging alone can drive neuronal degeneration through mechanisms including BBB deterioration and mitochondrial dysfunction [116,117]. Epidemiological data also indicate the age-dependent increase in the incidence and prevalence of PD [118]. Natural neuroactive compounds offer unique advantages due to their multi-targeted mechanisms. Clinical studies indicate that bioactive components in TCM can delay symptom onset by approximately 2.3 years [119]. Furthermore, the interplay between aging-related pathologies and the multi-targeted regulation of TCM forms a closed-loop intervention mechanism, offering novel pathways for PD prevention and treatment.

**Figure 8:**
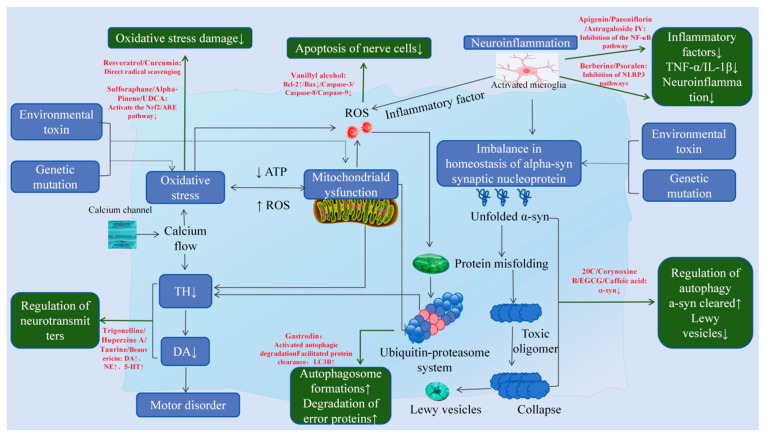
Common pathological mechanisms of PD and anti-PD effects of representative neuroactive and bioactive compounds from natural sources. The black arrows indicate the primary pathological mechanisms underlying PD development, the green arrows denote alterations in these mechanisms following exposure to key bioactive compounds, and the red text highlights representative bioactive compounds and their primary effects. White arrows within blue boxes indicate the up-regulation (↑) or down-regulation (↓) of targets in the pathological pathway. Red arrows represent drug-induced effects, denoting an increase (↑) or decrease (↓) in target abundance. White arrows within green boxes signify the enhancement (↑) or attenuation (↓) of pathway activity.

Clinical and mechanistic studies indicate that naturally derived neuroactive compounds exert multidimensional effects in PD, demonstrating significant efficacy in improving patients’ cognitive function, motor complications, sleep quality, and non-motor symptoms such as depression. Concurrently, they reduce adverse reactions associated with chemical drugs, thereby enhancing patients’ quality of life. Mechanistically, these compounds regulate inflammation-related pathways, namely NLRP3, NF-κB, and TLR4 signaling pathways, activate neuroprotective pathways including Nrf2/ARE and PINK1/Parkin, inhibit abnormal α-syn deposition, mitigate neuronal apoptosis, and improve mitochondrial dysfunction and oxidative stress. Collectively, these findings underscore their multi-target, multi-pathway neuroprotective potential (Figure 8).

This paper systematically reviews the significant potential of natural compounds to modulate multiple pathological pathways involved in PD. Many of these pathways overlap with those in other major neurodegenerative disorders of AD and amyotrophic lateral sclerosis (ALS). Shared mechanisms include pathological cross-talk between α-syn and Aβ/Tau, mitochondrial dysfunction, neuroinflammation, and apoptosis [120,121]. Therefore, the therapeutic compounds discussed in this paper may possess therapeutic relevance beyond PD, targeting a spectrum of neurodegenerative diseases with overlapping pathological features. Despite these promising preclinical findings, they can hardly be translated into clinically effective therapies capable of improving patient outcomes. Moreover, most natural extracts consist of chemically complex mixtures rather than single, well-defined compounds. This complexity poses fundamental challenges for identifying active constituents, establishing reliable quality control standards, and clarifying definitive component–effect–toxicity relationships. In addition, many structurally intricate natural products face severe bottlenecks in chemical synthesis and large-scale production, which constrain their standardized development and clinical translation. Further obstacles include low oral bioavailability, limited BBB penetration, insufficient long-term safety data in humans, and the complexity of designing and conducting large-scale clinical trials.

Addressing these challenges requires the implementation of biomarker-driven precision therapies. For instance, compounds targeting α-synuclein pathology or neuroinflammation should be prioritized for evaluation in patient subgroups harboring corresponding biomarkers (e.g., positive synuclein PET imaging, elevated levels of specific inflammatory factors). This biomarker-guided stratification may substantially improve the success rate of clinical trials [122]. Given that PD itself is a complicated network disease induced by the interplay of multiple pathological mechanisms, multi-target combination strategies hold broad prospects. Such strategies may involve co-administration of natural products with different mechanisms of action—for example, combining anti-inflammatory and antioxidant compounds—or integrating natural compounds with established standard therapies (e.g., levodopa) to address a broader spectrum of pathological processes and overcome the limitations of single-target interventions. Future research should further promote in-depth interdisciplinary integration, encompassing rational structural optimization through medicinal chemistry to improve compound properties and the development of advanced brain-targeted delivery systems to overcome the BBB. For example, advanced technologies such as fingerprint pattern analysis can enable comprehensive quality control throughout the production process. Successful paradigms like the Ginkgo biloba extract EGb 761 provide valuable references for developing and applying strictly standardized specific extracts. Although clinical exploration in this field remains limited, existing studies have provided important insights into the translational pathways [123]. The disease-modifying activities of lixisenatide (a GLP-1 receptor agonist) demonstrated in clinical trials validate the clinical feasibility of targeting specific pathways [124]. Similarly, clinical studies on mannitol have systematically evaluated natural products’ long-term safety and tolerability, which essentially advances the majority of preclinical active molecules toward human trials [125].

In summary, this review evaluates current clinical and preclinical evidence on neuroactive compounds with anti-PD potential, providing valuable insights for clinical application and highlighting promising pathways for future drug development. Future efforts should begin with in-depth mechanism studies, followed by rigorous pharmaceutical optimization and comprehensive safety assessments. Interdisciplinary collaboration and systematic research are essential to validate efficacy through biomarker-enriched precision clinical trials. Collectively, these strategies will further facilitate the translation of natural compounds into viable therapeutic strategies for Parkinson’s disease.

## Figures and Tables

**Table 1:** Representative drugs commonly used in clinical practice for treating PD and their adverse reactions.

Drug Type	Representative Drugs	Pharmacological Effects	Adverse Reactions
DA precursor drugs + dopa decarboxylase inhibitors	Levodopa-Benserazide	The inhibitor component reduces the peripheral conversion of levodopa to dopamine, allowing more levodopa to reach the brain and replenish striatal dopamine, thereby regulating the dopamine–acetylcholine balance.	Nausea, vomiting, orthostatic hypotension, abnormal involuntary body movements, depression, and difficulty urinating
MAO-B inhibitor	Selegiline Hydrochloride Tablets	Inhibits the action of monoamine oxidase in the body, thereby reducing the degradation of dopamine in the striatum.	Fatigue and dizziness
NMDA receptor antagonist	Amantadine	Antagonizes NMDA glutamate receptors; also promotes the release of dopamine from surviving neurons and may inhibit dopamine reuptake.	Hallucinations and mental confusion
Dopamine receptor agonist	Pramipexole	Mimics endogenous dopamine, activates dopamine receptor 2 in the nigrostriatal pathway	Nausea, drowsiness, hallucinations, and hypotension
Anticholinergic drugs	Benzhexol	Centrally antagonizes acetylcholine receptors, balancing the effects of dopamine and acetylcholine.	Anticholinergic reactions such as tachycardia, dry mouth, and constipation

## Data Availability

No new data were created or analyzed in this study. Data sharing is not applicable to this article.

## Abbreviations

The following abbreviations are used in this manuscript:

PD	Parkinson’s disease
SN	Substantia nigra
SNc	Substantia nigra pars compacta
α-syn	α-synuclein
DA	Dopamine
ACh	Acetylcholine
TH	Tyrosine hydroxylase
5-HT	5-Hydroxytryptamine
NE	Norepinephrine
GABA	Gamma-aminobutyric acid
AChE	Acetylcholinesterase
NMDAR	N-methyl-D-aspartate receptor
ROS	Reactive oxygen species
RNS	Reactive nitrogen species
LPS	Lipopolysaccharide
6-OHDA	6-Hydroxydopamine
PFFs	Pre-formed fibrils
LC3B	Microtubule-associated protein 1A/1B-light chain 3B
Bcl-2	B-cell lymphoma 2
Bax	Bcl-2-associated X protein
PI3K	Phosphoinositide 3-kinase
mTOR	Mammalian target of rapamycin
MAPK	Mitogen-activated protein kinase
p38 MAPK	p38 Mitogen-activated protein kinase
JNK	c-Jun N-terminal kinase
NF-κB	Nuclear factor kappa-light-chain-enhancer of activated B cells
IκB-α	Inhibitor of kappa B
TLR4	Toll-like receptor 4
MyD88	Myeloid differentiation primary response 88
TNF-α	Tumor necrosis factor-alpha
IL-1β	Interleukin-1 beta
IL-6	Interleukin-6
IL-4	Interleukin-4
IL-10	Interleukin-10
IL-17	Interleukin-17
IL-18	Interleukin-18
iNOS	Inducible nitric oxide synthase
COX-2	Cyclooxygenase-2
NLRP3	NACHT LRR and PYD domains-containing protein 3
CASP1	Caspase-1
GSDMD	Gasdermin D
PYCARD	PYD and CARD domain containing
Nrf2	Nuclear factor erythroid 2-related factor 2
ARE	Antioxidant response element
HO-1	Heme oxygenase-1
NQO1	NAD(P)H quinone dehydrogenase 1
GCL	Glutamate-cysteine ligase
GSH	Glutathione
GST	Glutathione S-transferase
GPx	Glutathione peroxidase
GR	Glutathione reductase
SOD	Superoxide dismutase
CAT	Catalase
MDA	Malondialdehyde
NO	Nitric oxide
PINK1	PTEN-induced kinase 1
Cyt c	Cytochrome c
mPTP	Mitochondrial permeability transition pore
Apaf-1	Apoptotic protease-activating factor 1
HMGB1	High mobility group box 1
SH-SY5Y	Human neuroblastoma cell line SH-SY5Y
H4	Human neuroglioma cell line H4
PC12	Rat pheochromocytoma cell line
BV2	Mouse microglial cell line BV2
MES23.5	Rat mesencephalic neuron x neuroblastoma hybrid cell line MES23.5
MN9D	Mouse dopaminergic neuron cell line MN9D
N2a	Mouse neuroblastoma cell line Neuro-2a
UPDRS	Unified Parkinson’s Disease Rating Scale
MoCA	Montreal Cognitive Assessment
EGCG	Epigallocatechin gallate
AS-IV	Astragaloside IV
BBR	Berberine
SFN	Sulforaphane
UDCA	Ursodeoxycholic acid
TUDCA	Tauroursodeoxycholic acid
GDEVs	Gardenia-derived extracellular vesicles
Cory B	Corynoxine B
ARIA-H	Amyloid-Related Imaging Abnormalities-Hemorrhage
MAO-B	Monoamine oxidase B
